# A comparative 30‐day outcome analysis of inpatient evaluation vs outpatient testing in patients presenting with chest pain in the high‐sensitivity troponin era. A propensity score matched case‐control retrospective study

**DOI:** 10.1002/clc.23435

**Published:** 2020-08-04

**Authors:** Osama Mahmoud, Hadi Mahmaljy, Hadi Elias, Edwin Hernandez Campoverde, Mohamed Youniss, Matthew Stanton, Katelyn Young, Maulin Patel, Rajesh Kuppuraju, Steven Jacobs, Insia Hashmi, Amro Alsaid

**Affiliations:** ^1^ Heart Institute, Geisinger Medical Center Danville Pennsylvania USA; ^2^ Department of Internal Medicine Geisinger Medical Center Danville Pennsylvania USA

**Keywords:** chest pain disposition, high‐sensitivity troponin, HEART score performance.

## Abstract

**Background:**

The best disposition of chest pain patients who rule out for myocardial infarction (MI) but have non‐low clinical risk scores in the high‐sensitivity troponin era is not well studied.

**Hypothesis:**

In carefully selected patients who rule out for MI, and have a high‐sensitivity troponin T ≤ 50 ng/L with an absolute increase less than 5 ng/L on repeat measurements, early emergency room (ER) discharge might be equivalent to inpatient evaluation in regards to 30‐day incidence of adverse cardiac events (ACEs) regardless of the clinical risk score.

**Methods:**

A total of 12 847 chest pain patients presenting to our health system ERs from January 2017 to September 2019 were retrospectively investigated. A propensity score matching algorithm was used to account for baseline differences between admitted and discharged cohorts. We then estimated and compared the incidence of 30‐day and 1‐year composite ACEs (MI, urgent revascularization, or cardiovascular death) between both groups. A multivariate Cox regression model was used to evaluate the effect of admission on outcomes.

**Results:**

A total of 2060 patients were matched in 1:1 fashion. The primary endpoint of 30‐day composite ACEs occurred in 0.6% and 0.4% of the admission and the discharged cohorts, respectively (*P* = .76). One‐year composite ACEs was also similar between both groups (4% vs 3.7%, *P* = .75). In a multivariate Cox regression model, the effect of inpatient evaluation was neutral (hazard ratio 1.1, confidence interval 0.62‐1.9, *P* = .75).

**Conclusions:**

Inpatient evaluation was not associated with better outcomes in our selected group of patients. Larger‐scale randomized trials are needed to confirm our findings.

## 1 INTRODUCTION

The advent of high‐sensitivity troponin (hsTn) assays has improved the identification of chest pain patients who can be safely discharged with low risk of adverse cardiac events (ACEs) compared to conventional assays^1^; however, the best disposition of chest pain patients who rule out for myocardial infarction (MI) by hsTn assays remains unclear when clinical risk scores are incorporated in the decision making process.

Although a considerable body of evidence has accumulated for the high negative predictive value of a normal hsTn result, the uncertainty stems from the widespread use of several risk estimation tools believed to be predictive of risk beyond the simple dichotomy of the results of cardiac biomarkers.^2^, ^3^, ^4^, ^5^ Indeed, clinical variables used in various risk estimation tools were found to be predictive of adverse outcomes independent of the cardiac‐specific troponin levels as measured via conventional assays.^6^ The performance of such risk estimation models has not been extensively studied in patients who rule out for MI in the hsTn era, however.^7^, ^8^, ^9^ Although major cardiology societies do not clearly recommend one management approach over another, it is a common practice to select an inpatient disposition for those with intermediate to high clinical risk scores who rule out for myocardial infarction.^10^, ^11^ It is not clear, however, if inpatient observation and testing are superior to outpatient management.

We intended to compare the outcomes of inpatient evaluation vs early emergency room (ER) discharge.

## 2 METHODS

### 2.1 Study design

This was a retrospective cohort study of patients with a primary or secondary diagnosis of chest pain presenting to any of Geisinger health system 12 acute care hospital emergency rooms in the period from January 2017 to September 2019. The study aims to compare the 30‐day incidence of ACEs in MI rule‐out patients undergoing inpatient vs outpatient evaluation. Patients were considered for inclusion if they met all of the following criteria: older than age 18, had at least two sets of high‐sensitivity troponin T (hsTnT) with the highest measurement being 50 ng/L or less, have ruled out for MI based on a flat troponin trend (absolute rise <5 ng/L),^10^ and had at least 30 days of follow‐up as defined by having an encounter with a Geisinger healthcare provider any time after 29 days of the index ER visit. Patients with a confirmed diagnosis of pulmonary embolism, aortic dissection, acute heart failure, or sepsis were excluded.

After obtaining institutional review board (IRB) approval, the electronic medical record (EMR) was queried to identify our study subjects as well as extract data regarding patients demographics, comorbidities, prior coronary revascularization, results of emergency room laboratory tests, ischemia evaluation within 30 days of the index ER visit including stress testing, coronary computed tomography angiography, and invasive coronary angiography. The electronic record was also searched for the occurrence of coronary revascularization, myocardial infarction, or death within 30 days and within 1‐year following the ER encounter. Chest pain characteristics and electrocardiography (EKG) results were obtained via manual chart review. Chest pain quality was decided based on treating clinicians' interpretation and, if not available, applying Diamond‐Forrester criteria.^12^ EKG results were categorized into normal findings, abnormal findings, and ischemic based on HEART score criteria.^3^

### 2.2 Endpoint definitions

The primary endpoint was the incidence of composite ACEs (urgent revascularization, MI, or cardiovascular death) within 30 days of the index ER visit. Secondary outcomes included the elements of the 30‐day composite, 30‐day all‐cause mortality, 1‐year composite ACEs, and 1‐year all‐cause mortality. Urgent coronary revascularization was defined as the occurrence of acute cardiac symptoms necessitating an ER or an urgent outpatient visit leading to hospital admission and the performance of a coronary revascularization procedure. Myocardial infarction was defined as per the fourth universal definition of spontaneous (type 1) MI.^13^ Cardiovascular death was defined as cardiac arrest secondary to an acute cardiac event or unexplained sudden death in patients without an active terminal condition. All outcomes were adjudicated via manual chart review with strict adherence to the aforementioned definitions.

### 2.3 Laboratory assessment

HsTnT was measured via Roche Diagnostic immunoassay (Roche Diagnostic, Mannheim, Germany). HsTnT results were used to stratify our cohort into three groups. Group 1 had HsTnT level < 6 ng/L, group 2 had levels between 6 ng/L and the sex‐specific 99th percentile upper reference limit (URL) (14 ng/L for females, 22 ng/L for males), and group 3 had levels between the 99th percentile URL and ≤ 50 ng/L. Since the FDA regulations prevent the reporting of results less than the limit of quantification, hsTnT levels below 6 ng/L is reported as <6 ng/L, although the limit of detection for the assay used is reported to be 3 ng/L.^14^

### 2.4 Statistical analysis

Data were summarized as numbers and proportions for categorical variables, as means ± SD for normally distributed continuous variables, and as median and interquartile range (IQR) for nonnormally distributed continuous variables. Group comparisons were carried out using Pearson chi‐square test or Fisher exact test for categorical variables as appropriate, and by independent sample *t*‐test, one‐way ANOVA, or Kruskal‐Wallis test for continuous variables. Kaplan‐Meier curves were used to express ACEs free survival and were compared using log‐rank test. Univariate and subsequently multivariate Cox regression were done to estimate hazard ratios of various demographic and clinical variables for predicting 1‐year ACE. A *P* value of ≤.1 for univariate regression of a predictor was used as a cutoff for inclusion in the multivariate model. Time censoring for Kaplan‐Meier and Cox regression was determined by time to last follow‐up date or time to event. Given the expected differences between the admitted and the discharged cohorts, we conducted a propensity score matching to account for the baseline differences in clinical risk. We estimated the propensity score for admission using admission as the dependent variable through a multivariable logistic regression model utilizing several clinical variables that included age, gender, history of diabetes mellitus, hypertension (hyperlipidemia), renal dysfunction, smoking, obesity, known coronary artery disease (CAD), prior MI, and prior coronary revascularization. A total of 4730 patients were matched in 1:1 fashion and manual chart review was performed to extract EKG findings, chest pain character, family history of premature CAD, left ventricular ejection fraction, stress testing and coronary angiography results, and the presence of exclusion criteria. Duo to residual unacceptable between‐group imbalances, additional propensity score matching based on EKG findings, chest pain characteristics, and heart score was conducted. We used a greedy neighbor 1:1 matching algorithm without applying a specific caliber of the propensity score yielding a sample of 2060 patients. We used a mean standardized difference (MSD) of 25% as a cutoff for small between groups imbalance for a given covariate.^15^ This MSD cutoff is more relaxed than the conventionally used 10%; however, it is considered acceptable.^15^, ^16^ The statistical software SPSS version 26 (IBM Corp., Armonk, New York) was used for analyses. Two‐sided *P*‐value for statistical significance was set at less than .05.

## 3 RESULTS

### 3.1 Patient characteristics

**FIGURE 1:**
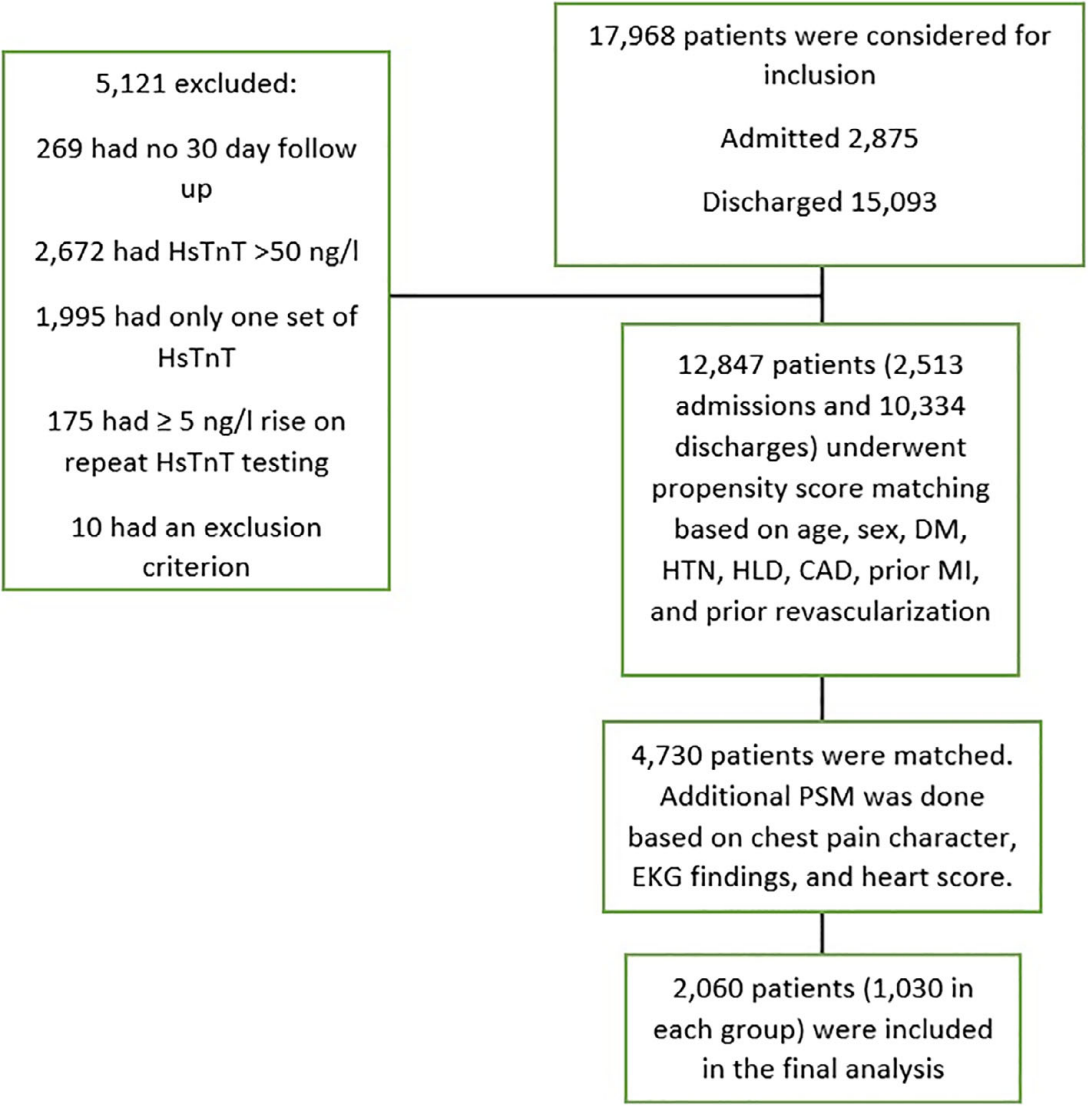
Flowchart of subjects' selection and exclusion. CAD, coronary artery disease; DM, diabetes mellitus; EKG, electrocardiography; HLD, hyperlipidemia; HsTnT, high‐sensitivity troponin T; HTN, hypertension; MI, myocardial infarction; Ng/L, nanogram/L; PSM, propensity score matching

A total of 256 247 ER visits between January 2017 and September 2019 were screened, of which 17 968 (7%) had a diagnosis of chest pain. Of the 12 847 patients that met the inclusion criteria, 1030 admitted patients were matched with 1030 discharged patients (Figure 1).

The mean age was 66 (IQR 56‐66), and females constituted approximately 51% of the study subjects. After matching, the groups were well balanced as defined by MSD less than 25% for a given covariate. Table 1 compares the baseline characteristics between admitted and discharged patients before and after matching. Most of the study subjects had atypical chest pain (66%), and more than half had intermediate HEART score (58%), and abnormal but non‐ischemic EKG findings (55%). The admitted cohort, unsurprisingly, were more likely to undergo ischemic testing (72% vs 17%), coronary angiography (15% vs 2%), and coronary revascularization (2.3% vs 0.4%) (Table 2).

**TABLE 1:** Baseline characteristics before and after matching

	Before matching				After matching			
Characteristics	Admitted	Discharged	MSD	*P*‐value	Admitted	Discharged	MSD	*P*‐value
Number of patients	2365	2365	NA	NA	1030	1030	NA	NA
Age, median (IQR)	64 (54‐74)	65 (55‐75)	−0.063	.012	64 (54‐73)	68 (58‐77)	−0.245	<.001
Female, n (%)	1221 (51.6)	1236 (52.3)	−0.013	.66	527 (51.2)	528 (51.3)	−0.002	.97
DM, n (%)	647 (27.4)	651 (27.5)	−0.004	.90	297 (28.8)	314 (30.5)	−0.036	.41
HTN, n (%)	1247 (52.7)	1271 (53.7)	−0.02	.49	537 (52.0)	585 (56.8)	−0.094	.034
HLD, n (%)	1168 (49.4)	1149 (48.6)	0.016	.58	489 (47.5)	538 (52.0)	−0.095	.031
Renal dysfunction, n (%)	440 (18.6)	459 (19.4)	−0.021	.48	201 (19.5)	248 (24.0)	−0.113	.012
Smoking, n (%)	1431 (60.5)	1457 (61.6)	−0.022	.44	645 (62.6)	624 (60.6)	0.042	.34
Obesity, n (%)	1288 (54.5)	1265 (53.5)	0.02	.50	569 (55)	563 (54.7)	0.012	.79
CAD, n (%)	915 (38.7)	949 (40.0)	−0.03	.31	389 (37.8)	465 (45.0)	−0.149	.001
Prior MI, n (%)	524 (22)	502 (21)	0.02	.44	221 (21.5)	254 (24.7)	−0.075	.084
Prior coronary revascularization, n (%)	478 (20)	522 (22)	0.045	.12	198 (19.0)	239 (23.0)	−0.091	.027
Chest pain character								
Noncardiac	408 (17.3)	1097 (46.4)	−0.77	<.001	196 (19.0)	196 (19.0)	0	1
Atypical	1549 (65.5)	1121 (47.4)	0.38	<.001	652 (63.3)	705 (68.4)	−0.11	.014
Typical	408 (17.3)	147 (6.2)	0.29	<.001	182 (17.7)	129 (12.5)	0.137	.001
Initial EKG								
Normal	929 (39.3)	964 (40.8)	−0.03	.29	383 (37)	377 (36.6)	0.012	.784
Abnormal	1266 (53.5)	1240 (52.4)	0.02	.45	565 (54.9)	577 (56.0)	−0.023	.63
Ischemic	170 (7.2)	161 (6.8)	0.015	.61	82 (8)	76 (7.4)	0.022	.62
Heart score, median (IQR)	5 (4–6)	4 (3–5)	NA	<.001	5 (4–6)	5 (4–6)	NA	.46
Low, n(%)	432 (18.3)	644 (27.0)	−0.23	<.001	121 (11.7)	126 (12.2)	−0.015	.74
Intermediate	1668 (70.5)	1557 (65.8)	0.103	.001	632 (61.4)	582 (56.5)	0.1	.025
High	265 (11.0)	164 (6.9)	0.135	<.001	277 (26.9)	322 (31.3)	−0.098	.029
Highest HsTnT ng/ml, median (IQR)	9 (5–17)	10 (5‐16)	NA	.28	9 (5‐15)	11 (7‐18)	NA	<.001
Undetectable	640 (27)	617 (26.0)	0.02	.45	277 (26.9)	211 (20.5)	0.155	.001
Less than 99th percentile URL	1143 (48.3)	1206 (51.0)	−0.05	.067	545 (47.0)	522 (49.3)	0.045	.31
Between 99th percentile URL and 50 ng/L	582 (24.6)	542 (22.9)	0.04	.17	208 (20.0)	297 (28.8)	−0.197	<.001
LVEF <40%, n (%)	77 (3.3)	69 (2.9)	0.019	.501	30 (2.9)	27 (2.6)	0.016	.69

Abbreviations: CAD, coronary artery disease; DM, diabetes mellitus; EKG, electrocardiogram; HLD, hyperlipidemia; HsTnT, high‐sensitivity troponin T; HTN, hypertension; IQR, interquartile range; LVEF, left ventricular ejection fraction; MI, myocardial infarction; MSD, mean standardized difference; Ng/L, nanogram per liter; URL, upper reference limit.

**TABLE 2:** 30‐day incidence of ischemic testing, coronary angiography, and revascularization

	Admitted	Discharged	*P*‐value
Ischemic testing within 30 days, n (%)	746 (72.4)	171 (16.6)	<.001
Negative	647 (86.7)	115 (67.3)	<.001
Equivocal	13 (1.7)	19 (11)	<.001
Positive or Fixed WMA	83 (11.1%)	33 (19.3%)	.004
Non urgent coronary angiography within 30 days, n (%)	155 (15)	21 (2)	<.001
Non urgent revascularization within 30 days, n (%)	24 (2.3)	4 (0.4)	<.001

Abbreviation: WMA, wall motion abnormality.

### 3.2 Outcomes

**FIGURE 2:**
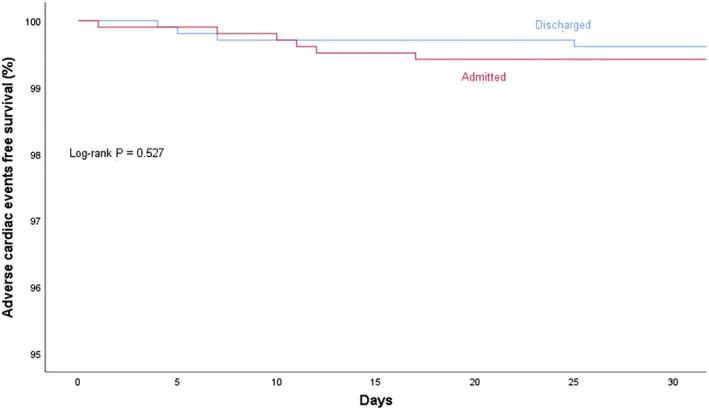
Kaplan‐Meier analysis for 30‐day adverse cardiac event‐free survival according to admission status

**FIGURE 3:**
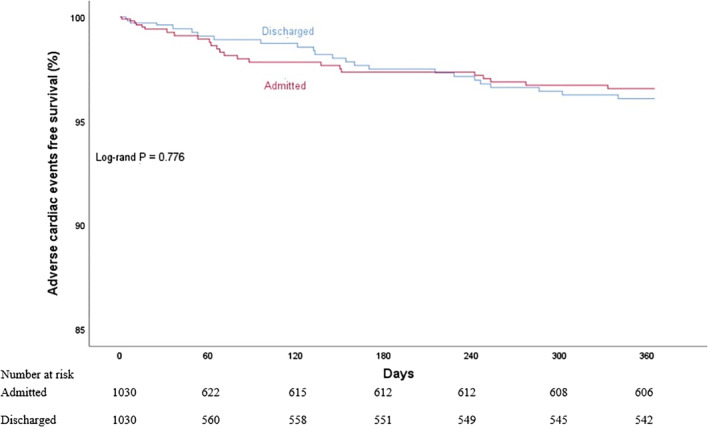
Kaplan‐Meier analyses for one‐year adverse cardiac events‐free survival according to admission status

There were no statistically significant differences in the primary endpoint of the 30‐day composite ACEs, or the individual components, between the admitted and the discharged groups. The same was observed for the rest of the secondary endpoints (Table S1). The event rates were strikingly low despite an overwhelming majority (88%) of patients scoring intermediate or high on the HEART score, which would predict a 6 weeks event rate of more than 16%.^17^ In a multivariate Cox proportional hazards regression model, neither admission nor HEART score was predictive of 1‐year composite ACEs. (Table S2). Kaplan‐Meier analyses for 30‐day as well as 1‐year composite ACEs free survival confirmed the lack of statistically significant differences between the groups (Log‐rank *P* = .527 and .776, respectively) (Figures 2 and 3).

In a post hoc sensitivity analysis involving the initial propensity score‐matched cohort (4730 patients) (Figure 1), the 30‐day composite ACE occurred in 18 (0.8%) patients in the early ER discharge cohort vs14 (0.6%) in the inpatient cohort (*P* = .48). The one‐year composite outcome was not statistically different as well (1.6% vs 1.9%, *P* = .38). In multivariate Cox regression models for prediction of the 30‐day and 1‐year composite outcomes, the adjusted hazards ratios of inpatient evaluation were 0.58 (CI 0.28‐1.2, *P* = .58) and 1.1 (CI 0.68‐1.8, *P* = .69) respectively, reaffirming a lack of benefit.

## 4 DISCUSSION

Numerous prognostication tools have been devised to aid in the management of chest pain patients ruled out for MI with the assumption that hospitalization would be a safer approach for those at a non‐low risk.^18^ The evidence that the risk is mitigable via an inpatient management approach is, however, scare. In a study utilizing Medicare data, inpatient evaluation seemed to result in fewer cases of MI and death as compared to a primarily outpatient management approach.^19^ On the contrary, it is well known that hospitalization, as well as cardiac testing, carry non‐trivial health risks to patients^20^, ^21^, ^22^ besides the unfavorable financial cost.^23^

The advent of hsTn assays has allowed faster‐triaging algorithms with strong evidence for a very low event rate when the level is below the 99th percentile URL for a given assay.^10^, ^24^ However, utilizing hsTn assays in clinical scoring algorithms, namely HEART score, does not seem to have a significant effect on the predictive value according to some analyses.^7^, ^8^, ^25^

Our study results are notable for two important observations. First, is the low short‐ and long‐term adverse event rates across both the study groups. It is important to emphasize, however, the selection criteria that excluded patients with an absolute hsTnT rise ≥5 ng/L, and those with hsTnT of more than 50 ng/L. Applying these hsTn rules seems to select for a group of patients at very low risk of adverse outcomes, even with 88% of our cohort having a HEART score in the intermediate or high‐risk categories. Furthermore, in this very select group of patients, the HEART score and the absolute hsTnT level were not predictive of the 1‐year composite ACE in a multivariate logistic Cox proportional hazards model. Second, there were no significant differences between the admitted and the discharged groups across all short‐ and long‐term outcomes despite the much higher utilization of stress testing, coronary angiography, and revascularization procedures in the admitted group.

Besides a relatively small sample size, our study bears several important limitations. First, the retrospective design carries a potential for underestimating event rates due to failure to capture events managed outside of the study institution. We do not believe this has affected our findings significantly for two reasons. Our institution is the largest local health network and health insurance provider in the area with a fairly stable population that is dependent on our network for their care. Also, we only included patients who had a visit with a provider at our institution at least 30 days after the index ER visit with the assumption that medical history will be updated at the time of the visit and an event that was treated at a different institution will still be captured in our EMR. Second, matching was challenging due to the inherent baseline differences between those who are decided for hospitalization and those who are discharged directly from the ER. Although some between‐group baseline differences exist, the effect size is estimated to be small. Furthermore, the discharged cohort seems to include relatively higher risk patients than the hospitalized cohort as evident by older age (median of 68 vs 65), a higher percentage of CAD (45% vs 38%), prior coronary revascularization (23% vs 19%), high‐risk HEART score (31.3% vs 26.9%), mild hsTnT elevation (28.8% vs 20%), and a smaller percentage of those with undetectable hsTnT (20.5% vs 26.9%). Third, data on medical treatments administered during the index encounter is lacking and we assumed that patients were treated according to standards of care. Lastly, the very low event rate and the relatively small sample size makes our study hypothesis‐generating rather than definitely conclusive. Large randomized prospective clinical trials are direly needed to shed more light on this critical topic.

## 5 CONCLUSION

An inpatient management approach does not seem to improve the outcomes of a carefully selected subgroup of chest pain patients with intermediate to high HEART scores, and hsTnT ≤50 ng/L with less than 5 ng/L increase on repeat measurement. These patients seem to be at a very low risk (<1%) of 30‐day ACEs regardless of the hospitalization status. Large‐scale randomized prospective clinical trials are needed to investigate further given the limitations of our study.

## CONFLICTS OF INTEREST

The authors declare no potential conflict of interest.

## Supporting information


**Appendix** **S1:** Supporting information
