# Supplementary material for: A comparative 30‐day outcome analysis of inpatient evaluation vs outpatient testing in patients presenting with chest pain in the high‐sensitivity troponin era. A propensity score matched case‐control retrospective study
Source: Clin Cardiol. 2020 Aug 4;43(11):1248–54. doi: 10.1002/clc.23435 (PMC7661656; doi:10.1002/clc.23435)
Supplement: Supplementary file 1 — Appendix S1: Supporting information [file CLC-43-1248-s001.docx]

| Outcomes | Admitted | | discharged | P-value | |
| --- | --- | --- | --- | --- | --- |
| Number | 1030 | | 1030 |  | |
| 30- Day outcomes | | | | | |
| ACE, n (%) ^a^ | 6 (0.6) | | 4 (0.4) | 0.76 | |
| MI, n (%) | 2 (0.2) | | 1 (0.1) | 1 | |
| Urgent revascularization, n (%) | 3 (0.3) | | 3 (0.3) | 1 | |
| Cardiovascular death, n (%) | 1 (0.1) | | 0 (0.0) | 1 | |
| All cause death, n (%) | 3 (0.3) | | 2 (0.2) | 1 | |
| 1-year outcomes | | | | | |
| Number | | 652 | 595 | |  |
| ACE, n (%) ^a^ | 24 (4.0) | | 24 (3.7) | 0.75 | |
| MI, n (%) | 11 (1.7) | | 11 (1.8) | 0.83 | |
| Urgent revascularization, n (%) | 9 (1.4) | | 9 (1.5) | 0.85 | |
| Cardiovascular death, n (%) | 4 (0.6) | | 6 (1.0) | 0.53 | |
| All cause death, n (%) | 28 (4.3) | | 34 (5.7) | 0.25 | |

Table S1.Comparison of 30-day and 1-year outcomes between admitted and discharged groups.
ACE: adverse cardiac events.
MI: myocardial infarct.
a: a patient who had more than one adverse cardiac event is counted only once in the composite incidence.

| Characteristics | | | | | Univariate | | Multivariate | |
| --- | --- | --- | --- | --- | --- | --- | --- | --- |
|  | | | | | **HR (95% CI)** | **P value** | **HR (95% CI)** | **P-value** |
| Age | | | | | 1.04 (1.01-1.06) | 0.003 | 1.01 (0.98-1.04) | 0.5 |
| Female | | | | | 0.6 (0.34-1.07) | 0.085 | 0.52 (0.28-0.95) | 0.032 |
| Obesity | | | | | 0.65 (0.37-1.2) | 0.14 |  |  |
| DM | | | | | 1.97 (1.1-3.5) | 0.019 | 1.5 (0.85-2.8) | 0.16 |
| HTN | | | | | 1.2 (0.64-2) | 0.67 |  |  |
| HLD | | | | | 0.83 (0.47-1.5) | 0.52 |  |  |
| Renal dysfunction | | | | | 2.4 (1.3-4.2) | 0.004 | 1.5 (0.77-2.9) | 0.24 |
| Smoking | | | | | 1.4 (0.78-2.6) | 0.255 |  |  |
| History of CAD | | | | | 2.2 (1.2-3.8) | 0.010 | 1.07 (0.53-2.2) | 0.85 |
| Prior MI | | | | | 1.8 (0.98-3.2) | 0.061 | 0.99 (0.49-2.0) | 0.98 |
| Prior coronary revascularization | | | | | 1.77 (0.97-3.2) | 0.64 |  |  |
| 30 days ischemia evaluation | | | | | 1.05 (0.6-1.9) | 0.88 |  |  |
| 30 days nonurgent revascularization | | | | | 1.8 (0.25-13) | 0.56 |  |  |
| Chest pain character | | | | |  |  |  |  |
|  | **Non-cardiac** | | | | Reference |  |  |  |
|  | **Atypical** | | | | 0.83 (0.39-1.8) | 0.63 |  |  |
|  | **Typical** | | | | 1.9 (0.8-4.4) | 0.149 |  |  |
| EKG findings | | | | |  |  |  |  |
|  | | | | **Normal** | Reference |  |  |  |
|  | | | | **Abnormal** | 2.6 (1.3-5.5) | 0.01 | 1.9 (0.87-3.9) | 0.108 |
|  | | | | **Ischemic** | 2 (0.63-6.6) | 0.24 |  |  |
| Heart score | | | | |  |  |  |  |
|  | | **Low score** | | | Reference |  |  |  |
|  | | **Intermediate** | | | 0.38 (0.21-0.71) | 0.002 | 4314 (0-7098) | 0.88 |
|  | | **High** | | | 3.9 (2.1-7.2) | <0.001 | 9090 (0-14980) | 0.87 |
| HsTnT results | | | | |  |  |  |  |
|  | | | **Undetectable HsTnT** | | Reference |  |  |  |
|  | | | **Less than 99^th^ percentile URL for gender** | | 1.2 (0.49-2.8) | 0.72 |  |  |
|  | | | **Between the 99^th^ percentile URL & 50 ng/l** | | 3.6 (1.6-8.4) | 0.003 | 1.5 (0.53-3.9) | 0.48 |
| Inpatient evaluation | | | | | 0.9 (0.53-1.6) | 0.78 | 1.1 (0.62-1.9) | 0.75 |

Table S2. Cox proportional univariate and multivariate hazard ratios of various variables for the one-year composite of myocardial infarction, urgent revascularization, or cardiovascular death.
HR: hazard ratio. CI: confidence interval. DM: diabetes mellitus. HTN: hypertension. HLD: hyperlipidemia. CAD: coronary artery disease. MI: myocardial infarction. HsTnT: high-sensitivity troponin T. Ng/l: nanogram per liter. URL: upper reference limit.
